# Aging-related motor function and dopaminergic neuronal loss in C57BL/6 mice

**DOI:** 10.1186/s13041-020-00585-6

**Published:** 2020-03-23

**Authors:** Sachiko Noda, Shigeto Sato, Takahiro Fukuda, Norihiro Tada, Nobutaka Hattori

**Affiliations:** 1 Department of Neurology, Juntendo University Graduate School of Medicine, Tokyo, 113-8421 Japan; 2 Division of Neuropathology, Department of Neuropathology, The Jikei University, School of Medicine, Tokyo, 105-8461 Japan; 3 Atopy Research Center, Juntendo University School of Medicine, Tokyo, 113-8421 Japan

**Keywords:** Dopaminergic neuron, C57BL/6 mouse, Aging, Mitochondria

## Abstract

Aging-related dopaminergic neuronal loss and its motor phenotypes are well known. Excessive loss of dopaminergic neurons leads to Parkinson’s disease (PD), the most common neurodegenerative disorder characterized by the loss of nigrostriatal dopamine–producing neurons. In mice, however, aging-related dopaminergic neuronal loss and its consequences for motor function are poorly understood. We observed the phenotype of wild-type C57BL/6 mice over an extended period of time. C57BL/6 mice exhibited age-dependent locomotor impairments, including hindlimb defects and the number of dopaminergic neurons decreased in aged mice, contributing to locomotor dysfunction. We observed a reduction in striatal dopamine levels in aged mice using high-performance liquid chromatography (HPLC). Thus, dopamine levels are affected by the loss of dopaminergic neurons. Furthermore, fragmented mitochondria were observed in dopaminergic neurons of aged mice but not in those of young mice. Aging-related dopaminergic neuronal loss and accumulation of damaged mitochondria may underlie the pathophysiology of aging.

## Main text

Mitochondrial dysfunction has been considered a major contributor to aging and age-related diseases. Mitochondrial dynamics change in advanced age; e.g., mitochondrial biogenesis decreases, while mitochondrial DNA damage and reactive oxygen species (ROS) production both increase [1, 2]. The main source of ROS is the mitochondria. The accumulation of ROS and oxidative damage have been linked to multiple pathologies, including neurodegenerative disorders, diabetes, cancer, and aging. Excessive ROS production is considered an important factor that accelerates the aging process [3, 4]. However, the phenotypes of aged mice have not been fully examined. To elucidate the critical role of aging in dopaminergic neurons, we characterized aging-related pathologies and decreases in motor function in C57BL/6 mice.

**Fig. 1:**
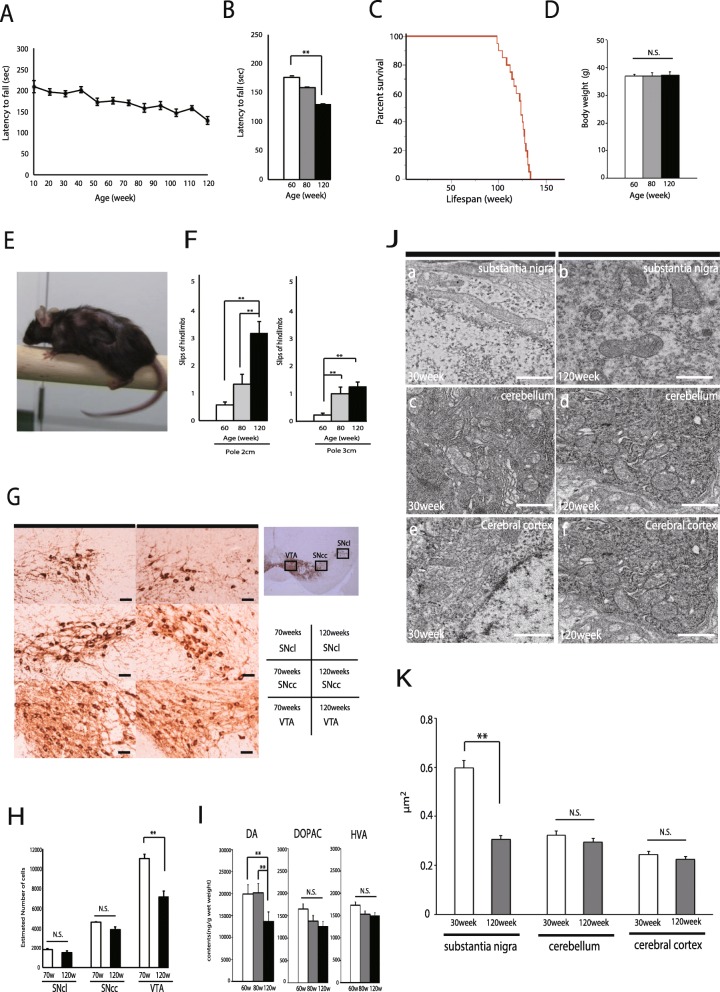
**a** In the accelerating rotarod assay, rotation was accelerated from 5 to 40 rpm over the course of 5 min, and fall latency was recorded. The experiments were performed using mice from 10 to 120 weeks of age (*n* = 70). Accelerating rotarod tests were performed on a rotarod machine with automatic timers and falling sensors (MK-660D, Muromachi Kikai, Japan). Data are means ± SE (error bars). **b** Quantification of fall latency. Aged mice experiments were performed using mice 60, 80, and 120 weeks of age. **c** Kaplan–Meier analysis of survival of C57BL/6 mice (*n* = 20). **d** Body weight of mice 60, 80, and 120 weeks of age (60-week-old mice, *n* = 10; 80-week-old mice, *n* = 10; 120-week-old mice, *n* = 10). Data are presented as means ± SE (error bars); ** *p* < 0.01 (ANOVA, Tukey post hoc pairwise comparisons). N.S.; Mice 60, 80, and 120 weeks of age denote not significant. **e** Runway test of aged-mice. The runway test was performed using a narrow horizontally fixed beam. Aged-mice could hardly move on the beam, and their hindpaws frequently slipped. **f** The number of hindlimb slips was recorded for 60-, 80-, and 120-week-old mice crossing the 2 cm (left) and 3 cm (right) pole. Data are presented as means ± SE (60-week-old mice, *n* = 10; 80-week-old mice, *n* = 10; 120-week-old mice, *n* = 10); statistical significance was evaluated using ANOVA, Tukey post hoc pairwise comparisons. ** *p* < 0.01. **g** Histological analyses of SN in 70-week-old mice and 120-week-old mice. Paraffin sections were immunostained for TH. SNcl, lateral area of the substantia nigra pars compacta; SNcc, center area of the substantia nigra pars compacta; VTA, the ventral tegmental area. Scale bars, 20 μm. **h** For stereological quantification, three areas were selected. Every other 40-μm section of serial coronal brain slices for each genotype was stained for DAB. Quantification was performed with design-based stereology system (Stereo-Investigator version 2019, MBF Bioscience, Williston, VT, USA). Sampling parameters were set up according to the software guide to achieve the coefficient of error ranged between 0.06 and 0.09 using the Gundersen test. Data are means ± SE (70-week-old mice, *n* = 5; 120-week-old mice, *n* = 18); ** *p* < 0.01 (Student’s *t*-test). N.S.; Not significant. **i** HPLC analysis of dopamine (left), dihydroxyphenylacetic acid (DOPAC) (middle), and homovanillic acid (HVA) (right) levels in the dorsal striatum of 60-, 80-, and 120-week-old mice. Data are presented as means ± SE (60-week-old mice, *n* = 5; 80-week-old mice, *n* = 5; 120-week-old mice, *n* = 10); ** *p* < 0.01 (ANOVA, Tukey post hoc pairwise comparisons). N.S.; Not significant. **j** For conventional electron microscopy, mice were fixed by cardiac perfusion with 2.5% glutaraldehyde in 0.1 mol/L PB (pH 7.2). Brain slices were embedded in epoxy resin, and ultrathin sections (70 nm thickness) were prepared and imaged on an HT7700 electron microscope (Hitachi, Japan). Electron micrographs of dopaminergic neurons in the SN (a,b), cerebellum (c,d), cerebral cortex (e,f); 30- (*n* = 3) (left) and 120-week-old mice (*n* = 3) (right). Scale bars, 1 μm. **k** Quantitation of mitochondrial area (30-week-old mouse dopaminergic, cerebellar, and cerebral cortical cells, *n* = 20; each 120-week-old mouse dopaminergic, cerebellar, and cerebral cortical cells, *n* = 20). The mean mitochondrial area in dopaminergic neurons was smaller in 120-week-old mice (right) than in 30-week-old mice (left). Significance was evaluated using Student’s *t*-test. * *p* < 0.01. N.S.; Not significant

We observed C57BL/6 mice over a longer period of time than in other studies. Fall latency during the accelerating rotarod test was reduced in aged mice (Fig. 1a). Latency to fall times were diminished at 120 weeks, compared with young mice (Fig. 1b). C57BL/6 mice were viable at birth and survived (Fig. 1c). And mean body weight was not changed (Fig. 1d). Moreover, in the runway test, young mice exhibited well-coordinated movement and almost no slips of either the forepaw or hindpaw from the beam. By contrast, aged mice could hardly move on the beam and slipped frequently (Fig. 1e, f). In particular, the hindpaws of mice at 120 weeks of age often slipped off the beam (Fig. 1f). To determine how decreasing TH neuron number contributes to aging, we compared the number of TH neurons between aged and younger mice. We sacrificed these mice (70-week-old mice, *n* = 5; 120-week-old mice, *n* = 18) and counted the number of TH neurons in three sections: the ventral tegmental area (VTA), the center area of the substantia nigra pars compacta (SNcc), and the lateral area of the substantia nigra pars compacta (SNcl). Mice at 120 weeks of age had fewer TH neurons than at 70 weeks of age. The reduction in TH cell number was most prominent in the VTA (Fig. 1g, h). No neuronal loss was observed in young mice that did not exhibit motor dysfunction (data not shown). The loss of dopaminergic neurons may contribute to the motor impairment observed in aged mice. In addition, we tested dopamine physiology in 120-week-old aged mice by neurochemical analysis of the dorsal striata. HPLC revealed a reduction in striatal dopamine levels and metabolites in aged mice relative to control mice (Fig. 1i). To further characterize these mitochondria, we performed ultrastructural analysis in dopaminergic neurons of 120-week-old mice. We observed small, round, and fragmented mitochondria in dopaminergic neurons in aged mice (Fig. 1Jb), but not in young mice (Fig. 1J a). Precise quantification revealed that mitochondria area was reduced in dopaminergic cells (Fig. 1k).

We found that no other studies thus far have observed normal aging while focusing on dopaminergic neurons in wild-type C57BL/6 mice. Several studies have demonstrated impaired neostriatal functioning due to senescence in rodents. Thus, it has been shown that striatal dopamine content, turnover and uptake [5–7] as well as the number of striatal dopamine receptors are reduced in the senescent male rodent [8, 9]. However, a few studies have attempted to systematically explore the link between these changes in striatal dopamine and motor behavioral deficits in aged mice. Furthermore, there have been no studies concerning dopaminergic neuronal loss in aged mice. In this report, we demonstrated age-dependent dopaminergic neuronal loss (Fig. 1g, h, i) and decrease in dopamine (Fig. 1h). About 35% in the VTA dopaminergic neurons was observed during normal aging. A balance of dopaminergic and cholinergic systems in the striatum has been suggested and imbalance between these two systems can result in movement disorders [10]. Under aging states in the dorsal striatum, an alteration in excitatory and inhibitory transmission modulated by neuromodulators such as dopamine may underlie dysfunctional locomotion. Aging-related neuronal loss has been reported in several areas of the brain. McGeer et al. found that the number of nigral neurons decreases in direct proportion to age, with 48% loss by the age of 60 years [11]. Osterburg et al. [7] reported that dopamine levels decrease within striatal regions in 24–30-month-old mice and C57BL/6 mice clearly do not undergo progressive dopamine loss between 3 and 21 months. Compared with humans, mouse dopaminergic neurons may be affected by aging past 100 weeks. Mitochondrial dysfunction has been considered a major contributor to aging and aging-related diseases. In aged C57BL/6 mice, dopaminergic neurons were filled with small, round mitochondria (Fig. 1j). The mean mitochondrial area was smaller than in younger mice (Fig. 1k). Fragmented mitochondria were also observed to accumulate in dopaminergic neurons of aged C57BL/6 mice. Previous studies have demonstrated that aging is accompanied by a decrease in mitochondrial dynamics that leads to compromised function and morphological alterations. In both the skeletal muscle and brain of aged individuals, mitochondria have been observed to be enlarged and more rounded [12–14]. On the other hand, Poggi et al. have reported a decrease in mitochondrial size in aged individuals [15]. Changes in mitochondrial volume, shape, and length seem to be a general feature of the human aging process.

The aging-related motor dysfunction and pathology we observed in aged C57BL/6 mice suggest that mitochondrial impairment may underlie aging. We found the accumulation of damaged mitochondria and dopaminergic neuronal loss in aged C57BL/6 mice. Although we require further examination to verify our findings, mitochondria may play a key role in the pathophysiology of aging and may be useful targets for preventing and treating chronic disease, as well as for promoting healthy aging.

## Data Availability

All data generated or analyzed during this study are included in this published article.

## Abbreviations

PD: Parkinson’s disease
HPLC: High-performance liquid chromatography
ROS: Reactive oxygen species
VTA: Ventral tegmental area
SN: Substantia nigra
SNcc: Center area of the substantia nigra pars compacta
SNcl: Lateral area of the substantia nigra pars compacta
DOPAC: Dihydroxyphenylacetic acid
HVA: Homovanillic acid
